# Sustainable deimplementation of continuous pulse oximetry monitoring in children hospitalized with bronchiolitis: study protocol for the Eliminating Monitor Overuse (EMO) type III effectiveness-deimplementation cluster-randomized trial

**DOI:** 10.1186/s13012-022-01246-z

**Published:** 2022-10-21

**Authors:** Christopher P. Bonafide, Rui Xiao, Amanda C. Schondelmeyer, Amy R. Pettit, Patrick W. Brady, Christopher P. Landrigan, Courtney Benjamin Wolk, Zuleyha Cidav, Halley Ruppel, Naveen Muthu, Nathaniel J. Williams, Enrique Schisterman, Canita R. Brent, Kimberly Albanowski, Rinad S. Beidas, for the Pediatric Research in Inpatient Settings (PRIS) Network, Prabi Rajbhandari, Emily Knuth, Michelle Bailey, Kate Lucey, Patty Stoeck, Samantha House, Alyssa Silver, Monique Naifeh, Michael Tchou, Amy Tyler, Vivian Lee, Erin Cummings, Clifton Lee, Kyrie Shomaker, Alexandra Mihalek, Courtney Solomon, Raymond Parlar-Chun, Kathleen Berg, Nick Ryan, Tina Halley, Mary Orr, Tracey Liljestrom, Erin Preloger, Padmavathy Parthasarathy, Rashida Shakir, Andrew Chu, Morgan Greenfield, Julianne Prasto, Ann Le, Kimberly Monroe, Andrea Lauffer, Meredith Carter, Kamilah Halmon, Glen Huff, Kiran Gadani Patel, Jennie Ono, Alan Schroeder, Gregory ( Greg) Plemmons, Michael Perry, Sumeet Banker, Jennifer Lee, Robert Willer, Begem Lee, Kyung Rhee, Richelle Baker, Polina Frolova Gregory, Vipul Parikh, Mini Wallace, Stephen Edwards, Lisa Beckner, Michelle Hamline, Lauren Solan, Leigh-Anne Cioffredi, Scarlett Johnson, John Andrake, Nicole Webb, Adam Berkwitt

**Affiliations:** 1 Section of Hospital Medicine, Children’s Hospital of Philadelphia, Children’s Hospital of Philadelphia Hub for Clinical Collaboration, 3500 Civic Center Blvd, Philadelphia, PA 19104 USA; 2 Center for Pediatric Clinical Effectiveness, Children’s Hospital of Philadelphia, 2716 South Street, Philadelphia, PA 19146 USA; 3 Department of Pediatrics, Perelman School of Medicine, University of Pennsylvania, Philadelphia, USA; 4 Penn Implementation Science Center at the Leonard Davis Institute of Health Economics (PISCE@LDI), University of Pennsylvania, Philadelphia, USA; 5 Department of Biostatistics, Epidemiology, and Informatics, Perelman School of Medicine, University of Pennsylvania, 206 Blockley Hall, 423 Guardian Drive, Philadelphia, PA 19104-6021 USA; 6 Department of Pediatrics, University of Cincinnati, Cincinnati, OH 45229 USA; 7 Division of Hospital Medicine, Cincinnati Children’s Hospital Medical Center, Cincinnati, USA; 8 James M. Anderson Center for Health Systems Excellence, Cincinnati Children’s Hospital Medical Center, 3333 Burnet Ave ML 9016, Cincinnati, OH 45229 USA; 9 Independent Consultant, Boston, USA; 10 Department of Pediatrics, University of Cincinnati College of Medicine, Cincinnati, USA; 11 Division of General Pediatrics, Boston Children’s Hospital, Enders 1, 300 Longwood Ave, Boston, MA 02115 USA; 12 Department of Pediatrics, Harvard Medical School, Boston, MA USA; 13 Department of Psychiatry, Perelman School of Medicine, University of Pennsylvania, 3535 Market Street, Philadelphia, PA 19104 USA; 14 Department of Medical Ethics and Health Policy, Perelman School of Medicine, Philadelphia, USA; 15 Leonard Davis Institute of Health Economics, University of Pennsylvania, Philadelphia, PA USA; 16 Department of Family and Community Health, School of Nursing, University of Pennsylvania, Philadelphia, USA; 17 Department of Biomedical and Health Informatics, Children’s Hospital of Philadelphia, 2716 South Street, Philadelphia, PA 19146 USA; 18 School of Social Work, Boise State University, 1910 W. University Drive, Boise, ID 83725 USA; 19 Institute for the Study of Behavioral Health and Addiction, Boise State University, Boise, USA; 20 Department of Medicine, Perelman School of Medicine, University of Pennsylvania, 3600 Civic Center Boulevard, 8th Floor, Philadelphia, PA 19104 USA; 21 Penn Medicine Nudge Unit, University of Pennsylvania Health System, Philadelphia, USA; 22 Center for Health Incentives and Behavioral Economics, Perelman School of Medicine, University of Pennsylvania, Philadelphia, USA; 23 Department of Medical Social Sciences, Feinberg School of Medicine, Northwestern University, Chicago, IL USA

**Keywords:** Bronchiolitis, Children, Deimplementation, Hospital, Infants, Lung, Nursing, Overuse, Pediatrics, Pulse oximetry

## Abstract

**Background:**

Methods of sustaining the deimplementation of overused medical practices (i.e., practices not supported by evidence) are understudied. In pediatric hospital medicine, continuous pulse oximetry monitoring of children with the common viral respiratory illness bronchiolitis is recommended only under specific circumstances. Three national guidelines discourage its use for children who are not receiving supplemental oxygen, but guideline-discordant practice (i.e., overuse) remains prevalent. A 6-hospital pilot of educational outreach with audit and feedback resulted in immediate reductions in overuse; however, the best strategies to optimize sustainment of deimplementation success are unknown.

**Methods:**

The Eliminating Monitor Overuse (EMO) trial will compare two deimplementation strategies in a hybrid type III effectiveness-deimplementation trial. This longitudinal cluster-randomized design will be conducted in Pediatric Research in Inpatient Settings (PRIS) Network hospitals and will include baseline measurement, active deimplementation, and sustainment phases. After a baseline measurement period, 16–19 hospitals will be randomized to a deimplementation strategy that targets unlearning (educational outreach with audit and feedback), and the other 16–19 will be randomized to a strategy that targets unlearning and substitution (adding an EHR-integrated clinical pathway decision support tool). The primary outcome is the sustainment of deimplementation in bronchiolitis patients who are not receiving any supplemental oxygen, analyzed as a longitudinal difference-in-differences comparison of overuse rates across study arms. Secondary outcomes include equity of deimplementation and the fidelity to, and cost of, each deimplementation strategy. To understand how the deimplementation strategies work, we will test hypothesized mechanisms of routinization (clinicians developing new routines supporting practice change) and institutionalization (embedding of practice change into existing organizational systems).

**Discussion:**

The EMO trial will advance the science of deimplementation by providing new insights into the processes, mechanisms, costs, and likelihood of sustained practice change using rigorously designed deimplementation strategies. The trial will also advance care for a high-incidence, costly pediatric lung disease.

**Trial registration:**

ClinicalTrials.gov,NCT05132322. Registered on November 10, 2021.

**Supplementary Information:**

The online version contains supplementary material available at 10.1186/s13012-022-01246-z.

Contributions to the literature

- Deimplementation of overused, ineffective interventions is essential to maximize quality and value and minimize harm, waste, and inefficiencies in the health care system.
- The science of deimplementation—and understanding of how to sustain deimplementation gains—is still evolving.
- The Eliminating Monitor Overuse trial will compare two strategies for deimplementing continuous pulse oximetry monitoring for a common pediatric respiratory illness, conduct a mechanistic evaluation of hypothesized mediators and moderators, evaluate deimplementation costs, and produce new knowledge to inform sustainable deimplementation of this and other overused, ineffective practices in pediatrics and across medicine.

## Background

Reducing the use of health interventions that are not supported by evidence is essential to maximize quality and value while minimizing harm, waste, and inefficiencies in health care [1, 2]. Medical overuse, as defined by the Institute of Medicine, is provision of care in the absence of a clear medical indication, or when the benefit does not outweigh the risk [3]. Overuse can be identified and measured when evidence-based guidelines specify conditions in which a practice is appropriate and also consider the balance between benefits and harms [4]. Deimplementation is the systematic and intentional reduction in overused practices that do not improve outcomes [5, 6]. In recent years, experts have called for deimplementation research to identify the best strategies for minimizing low-value care delivery, including in pediatrics [7, 8].

To investigate useful strategies to deimplement medical overuse, we focus on inpatient pediatric treatment of viral bronchiolitis (“bronchiolitis”), a common acute lung disease caused by a respiratory viral infection in children under 2 years old [9–11]. In the USA, bronchiolitis leads to over 100,000 hospitalizations annually [12]. Historically, this has occurred in a seasonal pattern, with most cases occurring between December and March [13]. Treatment typically includes feeding support, nasal suctioning, and in some situations supplemental oxygen [11]. Bronchiolitis patients are often continuously monitored with pulse oximetry (SpO_2_) despite evidence that it does not improve outcomes if used during periods of hospitalization when the patient is “in room air,” or not receiving supplemental oxygen [14]. Rather, in those patients, continuous SpO_2_ monitoring may identify brief, self-limited desaturations that do not require treatment and do not affect patient outcomes [15]. Overuse of continuous SpO_2_ monitoring is associated with increased oxygen administration, prolonged length of stay, unnecessary monitor alarms that can generate alarm fatigue, and increased costs [16–18]. Two clinical trials have demonstrated that intermittent SpO_2_ measurement is an equally safe alternative to continuous SpO_2_ monitoring for children in room air [19, 20], and three sets of national guidelines now discourage the use of continuous SpO_2_ monitoring in hospitalized children with bronchiolitis who are in room air [11, 21, 22]. Despite the evidence and guidelines, continuous SpO_2_ monitoring continues to be overused in hospitalized children with bronchiolitis, making it a prime target for deimplementation.

To prepare for the clinical trial outlined in this protocol, members of this authorship group conducted several preliminary studies. First, we conducted a 56-hospital, 3612-patient observational study of SpO_2_ monitoring and found that across all hospitals at baseline, 46% of bronchiolitis patients in room air were continuously SpO_2_-monitored, discordant with guidelines [14]. Furthermore, we found strikingly wide variation between hospitals, ranging from 2 to 92%, which was not attributable to differences in patient populations. This variation suggests that achieving guideline-concordant care is feasible, but the degree of success may be related to contextual factors. Second, we conducted qualitative interviews with clinicians and administrators from 12 hospitals to understand barriers to deimplementation, guided by the Consolidated Framework for Implementation Research [23, 24]. Key barriers included educational gaps, lack of clear instructions about when to monitor, and culture that normalizes monitoring. Third, we convened 39 stakeholders from 15 hospitals to develop deimplementation strategies using implementation mapping, a systematic approach to identifying strategies to address identified needs [25]. Applying Helfrich’s Dual Process Theory-Based Model for Deimplementation (described in detail under the “Frameworks and mechanisms” section below) [26], we categorized strategies into the following categories: (a) unlearning (educational outreach with audit and feedback [A&F]) and (b) substitution (replacing continuous monitoring with intermittent measurement, supported by a clinical pathway integrated into the electronic health record [EHR]). Fourth, we performed a 6-hospital, single-arm pilot trial of educational outreach with A&F, using historical controls. Each hospital improved compared to baseline, with mean rates of guideline-discordant care decreasing from 53 to 23% [27]. More than 90% of participating nurses and physicians also rated education and A&F to be feasible and acceptable deimplementation strategies [27]. Although the pilot trial showed immediate short-term success with deimplementation of unnecessary SpO_2_ monitoring, sustainment can be challenging; a systematic review of implementation studies found that less than half of the studies measuring sustainability outcomes reported successful sustainment more than 1 year after the initiatives to change practice [28]. Thus, further study of strategies that lead to successful sustainment is needed [29], especially given that no studies have examined the sustainment of deimplementation practice changes in pediatric hospital settings.

Building on this body of evidence, we will conduct the Eliminating Monitor Overuse (EMO) SpO_2_ trial, with study arms rooted in what we have learned from our observational studies and pilot trial. The trial will test the effects of an unlearning only strategy (educational outreach with A&F) compared to an unlearning + substitution strategy (educational outreach, A&F, and an EHR-integrated clinical pathway to encourage alternate recommended monitoring approaches) on the sustainment of deimplementation of SpO_2_ monitoring in children with bronchiolitis who are in room air. The trial will allow us to determine if the EHR-integrated clinical pathway enhances sustainment by continuing to support practice change, with a focus on its effects 1 year after withdrawal of educational outreach with A&F, and will offer insight into implementation strategy mechanisms. By focusing on an outcome (sustainment of deimplementation) that is highly relevant to both clinical practice and implementation science, we expect that trial results will have both clinical and scientific significance.

## Methods/design

This manuscript adheres to the Standard Protocol Items: Recommendations for Interventional Trials (SPIRIT) [30] and the CONSORT extension for cluster randomized trials [31] (Additional files 3 and 4).

### Trial management and protection of human subjects

The trial will be led by two principal investigators, CPB and RSB. Central management and regulatory coordination of the trial will be led by CRB and KA. Oversight of study operations and science will be provided by the Steering Committee of Co-Investigators, comprised of the remainder of the authors, plus two representatives from the National Heart, Lung, and Blood Institute: Aruna Natarajan, Program Director, Pediatric Lung Disease and Critical Care, and Karen Bienstock, Clinical Trials Specialist. The Data Coordinating Center will be housed at the Clinical Research Computing Unit at the University of Pennsylvania. Data entry will be done using Research Electronic Data Capture (REDCap) [32], with data validation checks performed in the electronic forms in real time and data quality check queries conducted by the Data Coordinating Center weekly. The Analytic Core will be housed at the Data Science and Biostatistics Unit at Children’s Hospital of Philadelphia. Data Use Agreements have been established with each participating site to regulate data flow and confidentiality procedures. A Data and Safety Monitoring Board (DSMB) has also been convened (Charter in Additional file 1). Site PIs at each participating site will provide direct oversight of local research activities.

This study was approved by the Institutional Review Board (IRB) at Children’s Hospital of Philadelphia. Prior to study commencement at each participating site, each US site established an IRB reliance agreement with Children’s Hospital of Philadelphia’s IRB using an electronic reliance platform. The Canadian site obtained local Research Ethics Board (REB) approval independently.

### Aims and hypotheses

This hybrid type III effectiveness-deimplementation cluster-randomized clinical trial includes three specific aims.

In aim 1, we will compare the effects of an unlearning only strategy (educational outreach with A&F) versus an unlearning + substitution strategy (educational outreach with A&F + an EHR-integrated clinical pathway) on the primary outcome of deimplementation sustainment and secondary outcomes at the hospital level, including equity, fidelity, and cost. Compared to the unlearning only strategy, we hypothesize that the unlearning + substitution strategy will result in better sustainment.

In aim 2, we will identify deimplementation strategy mechanisms linked to deimplementation outcomes using mixed methods, including questionnaires and qualitative interviews. Our mechanistic hypothesis is that the unlearning + substitution strategy will result in better deimplementation sustainment compared to the unlearning only strategy because the EHR-integrated clinical pathway will generate better routinization (clinicians developing new routines supporting practice change) and institutionalization (the organization embedding practice change into existing systems) of guideline-concordant care [33].

In aim 3, we will examine the effects of deimplementation on clinical outcomes and unintended consequences. We hypothesize that increased deimplementation penetration (i.e., a reduction in overuse of continuous monitoring) will be associated with decreased length of hospital stay for bronchiolitis. We will also perform active surveillance for underuse of continuous SpO_2_ monitoring in severely ill bronchiolitis patients as a potential unintended consequence of deimplementation.

### Trial overview

**Fig. 1:**
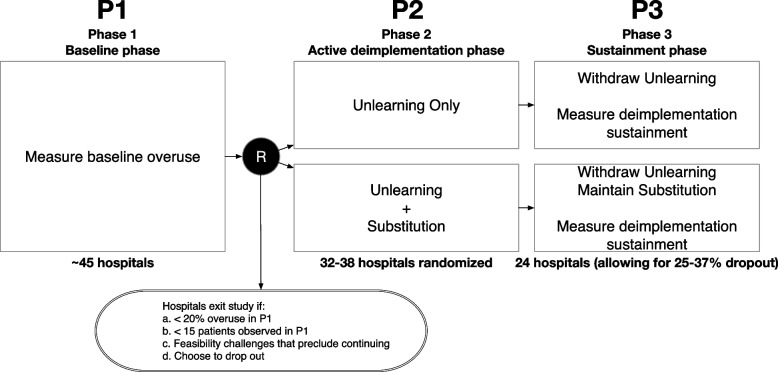
Trial diagram

As shown in Fig. 1, this hybrid type III effectiveness-deimplementation trial [34] with a longitudinal cluster-randomized design includes three main phases (baseline, active deimplementation, and sustainment). The unit of clustering is at the hospital level. Given the typical seasonal pattern of bronchiolitis, we originally designed each phase of the trial to take place during one of three winter periods (December–March). However, the COVID-19 pandemic and associated practices to reduce spread altered the typical seasonal pattern of viruses that cause bronchiolitis. Therefore, post-award, we revised the trial design, unlinking study phases from specific seasons with specific inter-phase durations described below.

#### Phase 1 (baseline, or P1)

We will measure baseline rates of overuse (guideline-discordant monitoring) in approximately 45 hospitals (see *Setting*). Based on this, we will exclude hospitals with data collection challenges (i.e., fewer than 15 patients observed for the presence or absence of continuous SpO_2_ monitoring overuse), those with low rates of overuse (i.e., less than 20%), and those with other feasibility challenges that preclude further participation. Hospitals may also elect not to continue in the trial after the baseline phase. We anticipate that this will result in 32–38 randomizable hospitals. If more than 38 hospitals remain after those exclusions, we will then randomize the 38 hospitals with the highest baseline rates of overuse. P1 was originally designed to occur over a 4-month period; however, due to low numbers of bronchiolitis patients in the first winter attributable to a seasonal shift in the incidence of respiratory viral disease (most notably the respiratory syncytial virus, RSV) [35], P1 was extended to a 7-month duration.

In the interim between P1 and P2, hospitals will have 6 months to prepare the deimplementation strategy rollout. This interim period may be extended beyond 6 months at the discretion of the Steering Committee in any of the following conditions: (a) ≥20% of sites in either arm are unprepared to start active deimplementation, (b) national RSV percent positivity is <2%, or (c) for other reasons, with DSMB and NHLBI approval.

#### Phase 2 (active deimplementation, or P2)

During this 4-month phase, deimplementation strategies will be deployed in the hospitals and overuse of continuous SpO_2_ monitoring will be simultaneously re-measured. At the end of P2, unlearning (educational outreach with A&F) will be withdrawn from both arms.

In the interim between P2 and P3 is a washout period that will last a minimum of 6 months and may be extended beyond 6 months at the discretion of the Steering Committee in any of the following conditions: (a) national RSV percent positivity is <2%, (b) the 4-month proposed phase 3 includes the month of July (coinciding with the arrival of new pediatric residents, a key stakeholder group), or (c) for other reasons, with DSMB and NHLBI approval.

#### Phase 3 (sustainment, or P3)

During this 4-month phase, the EHR-integrated pathway will be maintained exclusively in the unlearning + substitution arm. There will be no educational outreach or A&F in either arm. Overuse of continuous SpO_2_ monitoring will be re-measured and the primary outcome (deimplementation sustainment) will be contrasted between arms.

**Fig. 2:**
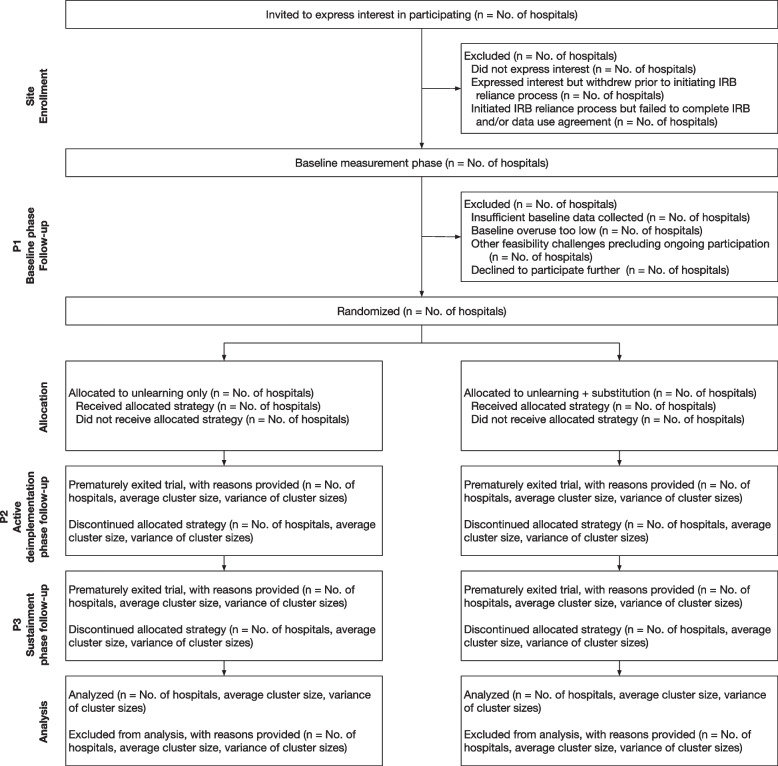
CONSORT diagram

Figure 2 provides a CONSORT diagram.

### Trial setting and hospital eligibility criteria

The trial will be conducted within Pediatric Research in Inpatient Settings (PRIS) Network hospitals [36]. PRIS is a 117-hospital research network whose Executive Council has experience leading high-impact studies of hospital care in children [37–40] and effectively sets the agenda for pediatric hospital medicine research nationally [10]. Sites participating in the EMO trial are listed on clinicaltrials.gov.

PRIS hospitals in the USA and Canada are eligible to participate in the trial. We will exclude sites that participated in our prior EMO observational study [14] but failed to collect sufficient data in that study to be included in the final analysis and/or had low baseline overuse.

### Study populations

#### Children with bronchiolitis

Our patient population includes children aged 2–23 months old with bronchiolitis who are hospitalized on non-intensive care, non-emergency department, non-step down inpatient units at participating hospitals. Bronchiolitis must be their primary diagnosis, and they must be cared for by a generalist inpatient service. Children with major comorbidities, those with COVID-19, and those born prior to 28 weeks will be excluded.

#### Parents or guardians of bronchiolitis patients

A subset of parents or guardians of bronchiolitis patients who were treated on a study hospital unit will participate in qualitative interviews. Interviews will focus on those who received treatment during the most recent study phase. Recruitment details are provided in the “Qualitative mechanistic measures” section below.

#### Hospital staff

A subset of hospital staff will complete study questionnaires and participate in qualitative interviews. Recruitment details are provided in the “Quantitative mechanistic measures” and “Qualitative mechanistic measures” sections below.

### Frameworks and mechanisms

As noted, Helfrich’s Dual Process Theory-Based Model for Deimplementation forms the theoretical basis for our experimental design [26]. Dual Process Theory specifies two types of reasoning underlying decisions. Type 1 reasoning is fast and intuitive [41, 42] and type 2 reasoning is analytical, slow, and resource-intensive [41, 42]. Helfrich’s framework separates deimplementation strategies into approaches that target each type of reasoning. Unlearning the ineffective practice using knowledge-based methods engages type 2 reasoning (e.g., presenting clinicians with evidence and guidelines, conducting A&F), and substituting the ineffective practice with an alternative practice supports type 1 thinking (e.g., using EHR-integrated clinical pathways). Helfrich’s model also highlights the importance of psychological reactance (a combination of negative emotion and cognition) that can occur in response to deimplementation efforts when freedom—in this case clinical autonomy—is perceived to be threatened [43].

We also draw on Slaghuis’s Framework for Sustainability of Work Practices, which complements Helfrich’s model [33] and posits that sustainment requires (a) routinization, whereby clinicians develop new routines such that the practice change becomes part of their everyday work, and (b) institutionalization, whereby the organization embeds the practice into its existing systems and structures via clinical protocols, policies, or pathways. In combination, these models explain the mechanisms behind our hypothesis that adding the substitution approach to the unlearning approach is expected to result in higher sustainment of deimplementation gains. The addition of the EHR-integrated clinical pathway will support routinization and institutionalization of the practice change while also harnessing type 1 reasoning to mediate the relationship between the EHR-integrated clinical pathway and deimplementation sustainment outcomes [33].

### Deimplementation strategies

All deimplementation strategies are assigned and delivered at the cluster (hospital) level.

#### Educational outreach (both trial arms)

Educational outreach to clinicians will focus on communicating core messages to staff, including the national guidelines, the evidence and rationale underlying the guidelines, and talking points to use if parents ask about monitoring, using language adapted from a parent-focused intervention [44]. Educational outreach will include several formats: (1) in-person sessions on each participating unit of each hospital prior to the start of active deimplementation, delivered in site-specific forums, with refresher sessions monthly for the remainder of the active deimplementation phase; (2) locally adapted handouts and posters; and (3) short educational videos and messaging distributed by email.

#### Audit and feedback (both trial arms)

A&F will follow our successful pilot study methods [27] and will include two levels: (1) weekly unit-level feedback and (2) real-time, clinician-level, inquiry-based feedback. Each week, we will compute the prior week’s percentage of bronchiolitis patients in room air who were inappropriately monitored continuously at the hospital and unit level and distribute these data to sites in the form of a visual dashboard that includes comparisons over time and between hospitals. Site PIs will then share the dashboards locally with clinicians in person (e.g., during staff meetings) and via email. Real-time 1:1 feedback will occur during clinical care; when collecting data on individual patients (as described below), data collectors encountering monitor overuse—continuous monitoring in a patient not receiving supplemental oxygen—are empowered to ask any available clinician responsible for that patient’s care, in a nonjudgmental way, about the indications for monitoring that patient.

#### Clinical pathway integrated into the EHR (substitution trial arm only)

The substitution strategy includes a clinical pathway integrated into the EHR, to guide clinicians step-by-step through guideline-concordant monitoring practices [45]. During year 1 of the trial, clinical stakeholders will participate in a guideline-to-pathway translation exercise. Based on the existing guidelines, the new pathway will clearly specify (a) situations when it is appropriate to initiate intermittent SpO_2_ measurement (the alternative practice) instead of continuous SpO_2_ monitoring and (b) when it is appropriate to discontinue continuous SpO_2_ monitoring and transition to intermittent SpO_2_ measurement.

Since integrating pathways into an EHR is a form of clinical decision support, we will incorporate the “Five Rights” of clinical decision support, which aim to ensure delivery of (1) the right information, (2) to the right people, (3) in the right intervention format, (4) through the right channels, (5) at the right point in the workflow (Table 1) [46, 47]. This will facilitate a standard approach to EHR integration while also allowing flexibility in format to encourage maximum feasibility and fit with local workflow [48, 49]. Following randomization, each site assigned to the unlearning + substitution arm will be matched with an EHR integration “coach” drawn from the Pediatric Clinical Decision Support Collaborative [50]. Each coach will facilitate integration of the clinical pathway into the local EHR by liaising directly with the Site PI, local clinicians, and informatics staff to ensure decision support that is aligned with the guiding principles in Table 1 is in place, on time, and within local capabilities.

**Table 1:** Guiding principles for EHR integration

Right information	When to initiate intermittent measurement instead of continuous monitoring When to transition from continuous monitoring to intermittent measurement after supplemental oxygen is discontinued
Right people	Clinicians who order monitoring Nurses who monitor patients for clinical changes and place or remove monitoring equipment
Right channels	EHR and Clinical Pathways Program webpage (via web link available exclusively to unlearning + substitution arm hospitals)
Right points in workflow	Appearing on screens, order sets, flowsheets, reports, and/or note templates used during hospital admission, supplemental oxygen management, and rounds
Right format(s) may include, but not be limited to, any combination of:	• EHR-embedded link to pathway website presented to clinicians at appropriate points in workflow (minimum standard) • Order set for bronchiolitis monitoring that guides clinicians to appropriately order (a) guideline-concordant monitoring initiation and (b) guideline-concordant transition to intermittent measurement, and that clearly communicates instructions to staff • Clinical reminder/alert that notifies nurses that continuous monitoring may no longer be indicated based on a documented discontinuation of supplemental oxygen

### Randomization

Hospitals eligible for randomization based on baseline measurement results will be cluster-randomized to either the *unlearning only* (anticipated *n*=19) or *unlearning + substitution* (anticipated *n*=19) arm. We will use covariate-constrained randomization methods [51] to achieve optimal balance between arms for three important hospital characteristics: (1) hospital type (freestanding children’s hospitals vs. general or community hospitals), (2) presence of pre-existing EHR clinical decision support for bronchiolitis that promotes the use of intermittent “spot checks” instead of continuous pulse oximetry in patients not requiring supplemental oxygen, and (3) baseline overuse rate. Randomization and assignment to study arms will be conducted by the Analytic Core overseen by the lead biostatistician (RX).

### Equitable deimplementation

We recognize that efforts to change clinical practice have the potential to inadvertently increase inequities [52]. Throughout the trial, our Data Coordinating Center will perform ongoing surveillance for signals in the data that may suggest hospital- or study-level inequities in deimplementation, with a focus on patient sex, race, and ethnicity (primarily contrasting non-Hispanic white with non-Hispanic Black and Hispanic patients), and preferred language of the patient’s family (primarily contrasting families who report a preference to communicate about their child’s health in a language other than English versus those who prefer English). If clinically significant signals are identified at any point, we will meet with the site PIs at affected hospitals promptly to discuss possible underlying reasons for the disparities and to develop mitigation plans with input from the study’s DSMB and Steering Committee [52].

### Study measures, procedures, and analysis

#### Deimplementation measures

##### Deimplementation sustainment/penetration

The primary outcome of deimplementation sustainment will be assessed as a longitudinal difference-in-differences in deimplementation penetration, or the extent to which the overused continuous SpO_2_ monitoring practice has been discontinued [53]. This will be captured by analyzing the change in the percentage of bronchiolitis patients who are in room air but are continuously SpO_2_-monitored across the 3 study phases. Because initiation and discontinuation decisions may differ from one another, we will assess penetration in two distinct categories of patients in room air: (1) those who never required supplemental oxygen and (2) those who previously did but subsequently stabilized. We will observe continuous SpO_2_ monitoring in order to measure this outcome, as we have done successfully in prior studies [14, 27], given that our prior research has shown that analyzing orders for monitoring does not accurately capture actual monitoring status [54]. Research staff at each hospital will perform cross-sectional observational data collection rounds during each phase. During these data collection rounds, trained research staff walk to the units of all eligible children with bronchiolitis and determine the continuous monitoring status of each patient based on visual examination of waveforms displayed on the monitor in each patient’s room or at a central monitoring station. In hospitals with direct integration of the monitors within the EHR or remote monitor viewing systems, visual examination of waveforms or parametric data may be performed using those platforms.

The primary analysis will be based on the intention-to-treat principle, with a secondary per-protocol analysis. We will analyze deimplementation sustainment as a longitudinal difference-in-differences comparison between study arms of the change in deimplementation penetration between the baseline phase (P1) and the sustainment phase (P3, after withdrawal of educational outreach with A&F), expressed as (P3-P1 | Arm 2) - (P3-P1 | Arm 1). We will use generalized hierarchical mixed-effects models with logit link for longitudinal binary outcome data. To account for differences in patient-level factors, we will adjust for patient age, gestational age, time since weaning from supplemental oxygen, presence of an enteral feeding tube, and whether data were captured during an overnight shift.

##### Sample size calculation based on deimplementation sustainment

The trial’s overall power analysis is based upon the primary outcome (deimplementation sustainment). The sample size is primarily driven by the number of hospitals, the within-hospital correlation over time, and the variation across hospitals. The degree of correlation can be expressed as either the intra-cluster correlation coefficient or the between-cluster coefficient of variation [55]. While the two approaches are equally valid, we have used the between-cluster coefficient of variation method in our calculations because it is more flexible and is more readily understood [55, 56]. Based on our preliminary studies, we estimate 50% deimplementation penetration (i.e., 50% overuse) at baseline (in P1). With 2-sided alpha = .05, moderate within-hospital correlation across phases of 0.6, and moderate to high between-hospital standard deviation of 15 percentage points, we will have 80% power to detect a difference of 16 percentage points between study arms if 24 total hospitals (12 per arm) complete the active deimplementation and sustainment phases (P2 and P3). Challenges related to the COVID-19 pandemic and disruption of the normal bronchiolitis seasonality prompted us to take a conservative approach to choosing the number of sites to randomize, accounting for the potential for unexpectedly high dropout between randomization and the end of P3. If 38 hospitals are randomized, our calculations allow for 37% dropout over the course of the trial.

##### Other deimplementation measures

Acceptability among nurses and physicians will be captured using the Acceptability of Intervention Measure (AIM) during the active deimplementation phase [57]. Site PIs will each facilitate the distribution of questionnaires to nurses and physicians who provide care for bronchiolitis patients on the units participating in the study, as well as to hospital administrators. Deimplementation fidelity will be captured as the extent to which educational outreach, A&F, and the EHR-integrated pathways are performed per protocol during active deimplementation. Fidelity data for educational outreach with A&F will be extracted from intervention logs maintained by Site PIs (e.g., to capture whether meetings happened as planned). Fidelity data for the EHR-based clinical decision support tool will be assessed using local EHR screenshots taken during the active deimplementation phase in order to assess alignment of the actual EHR interface with each guiding principle in the “Five Rights” framework. This fidelity assessment will focus on function rather than form, given that form is expected to vary. The cost of delivering each of the strategies will be assessed during the active deimplementation and sustainment phases, using the time-driven activity-based costing method [58].

#### Quantitative mechanistic measures

Using the same distribution methods and participants as described for the acceptability questionnaire above, we will administer questionnaires to assess hypothesized mediators and moderators. We will distribute the Slaghuis Measurement Instrument for Sustainability of Work Practices [33] to eligible clinical staff to assess potential mediators at two time points: following randomization and again in the final month of the sustainment phase, when we would expect the hypothesized mechanisms associated with sustainment to have occurred. The instrument assesses two closely related but conceptually distinct processes: *routinization*, in which clinicians develop new routines such that the practice change becomes part of their everyday work, and *institutionalization*, in which the organization embeds the practice change into its existing systems and structures. We will capture potential moderators during the active deimplementation phase, using the *Implementation Climate Scale* to understand whether hospital clinicians and staff perceive that they are expected, supported, and rewarded for deimplementation of continuous SpO_2_ monitoring [59] and the *Implementation Leadership Scale* to understand leader behaviors with regard to SpO_2_ monitoring [60]. We will also measure *psychological reactance* in clinicians using the same multiple choice instruments used in seminal reactance work to assess perceptions of threats to freedom in response to deimplementation messaging, emotional responses, and cognitive responses [61–63].

**Fig. 3:**
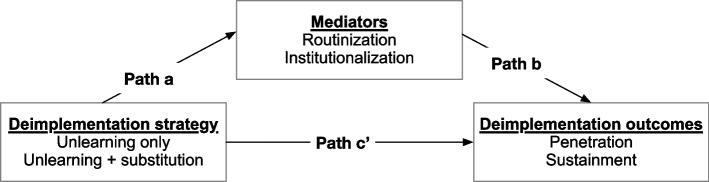
Mediation model

Mediation analysis will allow us to separate the direct effects of the exposure from effects that occur via an intermediate variable (indirect effects) [64]. For each outcome, we will perform separate mediation analyses for the routinization and institutionalization dimensions of the Slaghuis questionnaire [33]. Mediation will be tested using the product of coefficients approach [65–67], which we have used in previous studies [68–70]. In this approach, the total effect of the deimplementation strategy is parsed into direct and indirect effects. As shown in Fig. 3, path a represents the effect of the deimplementation strategy on the hospital-level mediators. Path b represents the effect of the hospital-level mediators on the outcomes. An unbiased estimate of the indirect mediated effect is derived via the product of the a and b paths [66, 67, 69]. Moderators (implementation climate, implementation leadership, and psychological reactance) will be tested separately by adding terms for each moderator and its interaction with the deimplementation strategy to the aim 1 models.

#### Qualitative mechanistic measures

Our qualitative inquiry aims to better understand mechanisms of practice change and potential effects on parents and guardians.

##### Hospital staff

Using a deviance case sampling approach [71, 72], we will conduct 48 semi-structured interviews with nurses and physicians who provide care to bronchiolitis patients in hospitals with the lowest and highest sustainment. Eligible clinicians will be identified at random from staff rosters and invited to participate in interviews to discuss their experiences related to the process of deimplementation and to explore mechanistic relationships between (a) the strategies, (b) quantitative study findings, and (c) sustainment.

##### Parents

We will conduct 15 semi-structured interviews with parents or guardians of children hospitalized with bronchiolitis who were found to be continuously SpO_2_-monitored while in room air during aim 1 data collection. Eligible parents or guardians will be identified at random from trial records during the sustainment phase and invited to interview by telephone within 4 weeks following discharge to explore their perceptions of, and reactions to, continuous SpO_2_ monitoring deimplementation.

Qualitative analysis will follow an integrated approach using the Consolidated Framework for Implementation Research as a starting framework while also allowing new concepts to emerge and become part of the coding scheme [73]. Our approach to integrating qualitative data with the quantitative data from Aim 2 will follow a “QUAN → qual” structure, where the function is to expand upon the quantitative findings to understand strategy mechanisms and the stakeholder perspectives on deimplementation efforts, and where the process is connecting [74]. We will use the quantitative data to identify patterns in the qualitative data by entering quantitative findings into NVivo as attributes of each participant. These attributes will be used to compare important themes among subgroups.

#### Clinical measures

To examine the effects of deimplementation on clinical outcomes and unintended consequences, we will measure the primary clinical outcome of length of hospital stay in hours and the secondary clinical outcome of oxygen supplementation duration in hours among enrolled bronchiolitis patients. We will also collect additional data to capture any underuse of monitoring that could plausibly occur in response to deimplementation in patients with more severe diseases [75]. We define underuse as failing to continuously monitor bronchiolitis patients receiving ≥2L/min supplemental oxygen or flow (a marker of more severe disease) [20] and will measure it using the same observational data collection methods used for the primary outcome. We will perform surveillance for additional unintended safety consequences [1]: code blue and rapid response team activations in bronchiolitis patients who were unmonitored at the time of the event and were subsequently found to be hypoxemic and (2) readmission of bronchiolitis patients to the hospital within 7 days of discharge with a finding of hypoxemia upon re-presentation to the emergency department. These outcomes will be extracted from charts and local patient safety databases.

In analyzing the clinical outcomes, hospital-level deimplementation penetration for each study phase will be the primary exposure variable. Hospital-level deimplementation penetration will be merged with patient-level length of stay and duration of oxygen supplementation. We will use generalized mixed-effects regression models to model the length of stay and duration of oxygen supplementation and use hospital-specific random intercepts to account for within-hospital clustering [76]. We will examine the underuse of continuous SpO_2_ monitoring during each study phase as the percentage of patients with bronchiolitis observed receiving ≥2L/min oxygen who are inappropriately unmonitored. We will analyze underuse longitudinally and by study arm using similar patient-level mixed effects logistic regression models as in the primary analysis.

#### Data sharing

After all participant enrollment has been completed, the Data Coordinating Center will prepare a final study database that has been stripped of identifiers for sharing. We will make the data available to users only under a data sharing agreement that provides for (1) a commitment to using the data only for research purposes and not to identify any individual participant, (2) IRB approval, (3) a commitment to securing the data using appropriate computer technology, and (4) a commitment to and an agreed-upon plan for destroying the data after analyses are completed. A plan to disseminate the findings is available in Additional file 2.

## Discussion

To our knowledge, the Eliminating Monitor Overuse (EMO) SpO_2_ trial will be the first in the field of pediatric hospital medicine to use a hybrid type III design to evaluate the comparative utility of two active strategies targeting sustained deimplementation of an overused practice. This trial builds upon our prior work, which demonstrated that about half of hospitalized children with bronchiolitis are monitored unnecessarily but also established that clinical practice can be quickly aligned with guidelines using educational outreach with A&F. This trial will allow us to determine whether the short-term gains we observed in our pilot trial are able to be sustained over time and compare alternative approaches to reaching sustainment that can inform the field of implementation science beyond our particular clinical focus in this trial.

Our study design has several strengths. First, we compare our combined strategy of educational outreach, A&F, and EHR-based clinical decision support to a common approach to clinical practice change and quality improvement in pediatric hospital medicine (educational outreach with A&F alone) [77–79], in keeping with both a National Heart Lung and Blood Institute Implementation Science Work Group conclusion that educational outreach and A&F are generally effective in improving outcomes [80], and with calls from experts to test A&F alone vs. A&F + co-interventions [81, 82]. Second, our chosen deimplementation strategies are based in theory (i.e., Helfrich’s Dual Process Theory-based deimplementation framework [26], Slaghuis’s Framework for Sustainability of Work Practices [33]) and consistent with evidence that multicomponent approaches have the greatest potential for success when aiming to reduce low-value care, that education is necessary but rarely sufficient, and that A&F and EHR-based clinical decision support approaches are the most promising strategies to address medical overuse [83–85]. In addition, our use of an EHR integration coach assisting each participating hospital goes beyond typical clinical decision support efforts and is meant to ensure that workflow prompts are optimized for the local context. Finally, we include family perspectives in our qualitative inquiry, in recognition of the fact that understanding their experiences is essential for achieving patient-centered care.

We also note several limitations. First, the pandemic and associated measures to reduce the spread of COVID-19 disrupted the well-established seasonal patterns of bronchiolitis disease [35], which led us to reconfigure study phases. The uncertainty of whether, and when, bronchiolitis will revert to being a disease confined to the winter months may threaten the ability to complete the trial as planned and may demand additional trial modifications. Second, it is possible that some hospitals in the unlearning only arm could develop EHR-based clinical decision support related to SpO_2_ monitoring during the trial period, leading to contamination between conditions, although this is discouraged.

In summary, the EMO SpO_2_ trial will advance the science of deimplementation, an understudied area of implementation science, by providing new insights from a pediatric research network into the processes, mechanisms, costs, and sustainment of rigorously designed deimplementation strategies. The trial will also advance pediatric hospital care for a high-incidence, costly pediatric lung disease that hospitalizes over 100,000 children annually.

## Supplementary Information

**Additional file 1.** DSMB Charter, Version Dated September 14, 2021. Description: Charter outlining the roles, responsibilities, practices, and procedures of the EMO Trial Data and Safety Monitoring Board.

**Additional file 2.** Dissemination plan for results of the EMO Trial. Description: Description of the plan for disseminating results and products of the EMO trial. This document was also submitted with the EMO Trial grant proposal.

**Additional file 3.** CONSORT 2010 checklist

**Additional file 4.** SPIRIT 2013 Checklist

## Data Availability

Not applicable.

## Abbreviations

A&F: Audit and feedback
AIM: Acceptability of Implementation Measure
CONSORT: Consolidated Standards of Reporting Trials
COVID-19: Novel coronavirus 2019
DSMB: Data and Safety Monitoring Board
EHR: Electronic health record
EMO: Eliminating Monitor Overuse
HIPAA: Health Insurance Portability and Accountability Act of 1996
IRB: Institutional Review Board
NHLBI: National Heart, Lung, and Blood Institute
NIH: National Institutes of Health
P1, 2, 3: Study phase 1, 2, or 3
PI: Principal investigator
PRIS: Pediatric Research in Inpatient Settings
REB: Research Ethics Board
REDCap: Research Electronic Data Capture
RSV: Respiratory syncytial virus
SPIRIT: Standard Protocol Items: Recommendations for Interventional Trials
SpO_2_: Pulse oximetry
US: United States
