# Supplementary material for: Sustainable deimplementation of continuous pulse oximetry monitoring in children hospitalized with bronchiolitis: study protocol for the Eliminating Monitor Overuse (EMO) type III effectiveness-deimplementation cluster-randomized trial
Source: Implement Sci. 2022 Oct 21;17:72. doi: 10.1186/s13012-022-01246-z (PMC9587657; doi:10.1186/s13012-022-01246-z)
Supplement: Supplementary file 1 — Additional file 1. DSMB Charter, Version Dated September 14, 2021. Description: Charter outlining the roles, responsibilities, practices, and procedures of the EMO Trial Data and Safety Monitoring Board. [file 13012_2022_1246_MOESM1_ESM.pdf]

Data and Safety Monitoring Board (DSMB) Charter

Study Name: The EMO Trial

Protocol Title: Eliminating Monitor Overuse (EMO) Hybrid Effectiveness-Deimplementation Trial

CHOP IRB Number: 21-018560

Funding: NHLBI U01 HL159880

Co-Principal Investigators:

Chris Bonafide, MD, MSCE
Children's Hospital of Philadelphia
Buerger Center for Advanced Pediatric Care
3500 Civic Center Blvd, 12th Floor
Philadelphia, PA 19104
Office Phone: 267-426-2901
email: bonafide@chop.edu

Rinad Beidas, PhD
University of Pennsylvania
3535 Market Street, 3rd floor, Room 3015
Philadelphia, PA 19104
Office Phone: 215-746-1759
email: rinad.beidas@pennmedicine.upenn.edu

Version Date: September 14, 2021

Table of Contents

1. Introduction 2
2. Responsibilities of the DSMB 2
3. Communication Plan 2
4. DSMB Membership 4
5. Scheduling, Timing, and Organization of Meetings 4
6. Organization of Meetings 5
7. Adverse Event Surveillance, Reporting, and Management 6
8. Summary Data Reports to the DSMB 8
9. Board Recommendations 9
10. Statistical Monitoring Guidelines 9
11. Appendix: DSMB Members 10

1. Introduction
This Charter is for the Data and Safety Monitoring Board (DSMB) for the Eliminating Monitor Overuse (EMO) Hybrid Effectiveness-Deimplementation Trial.

This Charter will be reviewed annually and updated as needed.

This hybrid effectiveness-deimplementation trial will be conducted in PRIS Network hospitals that care for children with bronchiolitis. Populations from which we will recruit include children with bronchiolitis, parents or guardians of bronchiolitis patients who participate in qualitative interviews, and hospital staff who care for bronchiolitis patients and participate in questionnaires and interviews. The University of Pennsylvania's Clinical Research Computing Unit (CRCU) serves as the Data Coordinating Center (DCC) for this study. Children's Hospital of Philadelphia (CHOP) serves as the reviewing Institutional Review Board (IRB). This is an unblinded trial, and investigators will not be blinded to results by treatment arm.

2. Responsibilities of the DSMB
The DSMB is responsible for safeguarding the interests of study participants, assessing the safety and efficacy of study procedures, and for monitoring the overall conduct of the study. In this role, the DSMB will review safety data, clinical outcome data, and implementation outcome data in each meeting that follows enrollment of the first subject.

The DSMB is an independent group advisory to the investigators and affiliated institutions. The DSMB is tasked with making recommendations about:

- Participant safety and risk/benefit ratio of study procedures and interventions, including whether new data from other sources affects the study
- Initial approval of the protocol and consent documents
- Methods to recruit participants
- Completeness, quality, and planned analysis of data
- Performance of individual centers

3. Communication Plan
The following diagram and description that follows illustrate the flow of information between the DSMB and other entities in this study. A co-investigator on the study team will be appointed as the DSMB Scientific Liaison. The DSMB Scientific Liaison will work with the MPIs, the CHOP-based Research Operations Project Manager, and the University of Pennsylvania DCC Project Manager to coordinate communication to the DSMB.

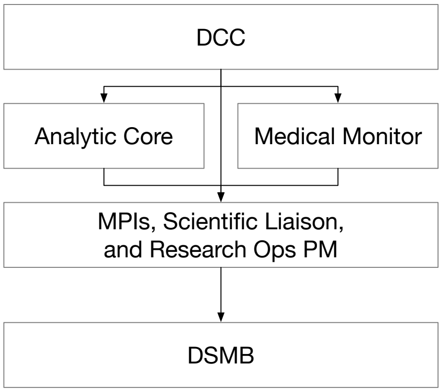

Descriptive data reports will be prepared by the DCC, sent to the MPIs, DSMB Scientific Liaison, and Research Operations Project Manager, who will then share it with the DSMB.

Data for interim and final analyses will be prepared by the DCC, deidentified, and shared with the Analytic Core. The analytic core will perform the analyses and share a report of results with the MPIs, DSMB Scientific Liaison and Research Operations Project Manager, who will then share it with the DSMB.

Adverse Event and Unanticipated Problem reports received by the DCC will be shared with the MPIs, Medical Monitor, Scientific Liaison, and Research Operations Project Manager. The Medical Monitor will assess all events reported by site investigators, reviewing and electronically signing each report. The monitor will then follow the CHOP IRB “Unanticipated Problems Decision Tree” (see figure below) and CHOP IRB SOP 408 “Unanticipated Problems” (available at https://irb.research.chop.edu/sites/default/files/documents/irbsop408.pdf) to determine the appropriate course of action.

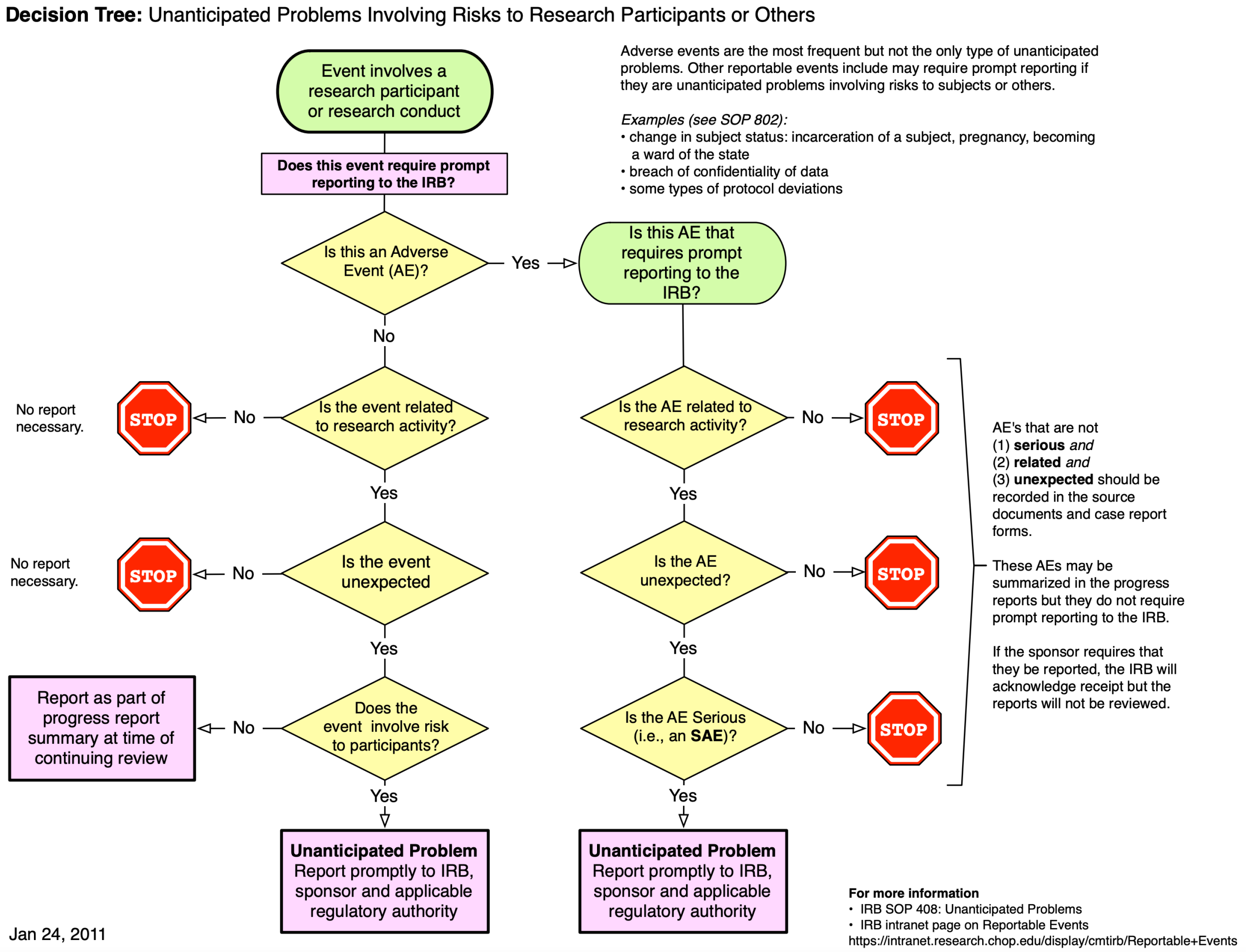

CHOP IRB Decision Tree for classification of unanticipated problems. The unanticipated problems in the lower right purple box are unanticipated problems that are SAEs. The unanticipated problems in the lower left purple box are unanticipated problems that are not AEs, but still represent risk to participants.

Definition of an Unanticipated Problem: According to the HHS Office for Human Research Protections, an unanticipated problem is any incident, experience, or outcome that meets all of the following criteria:

- Unexpected (in terms of nature, severity, or frequency) given (a) the research procedures that are described in the protocol-related documents, such as the IRB-approved research protocol and informed consent document; and (b) the characteristics of the subject population being studied;
- Related or possibly related to participation in the research (possibly related means there is a reasonable possibility that the incident, experience, or outcome may have been caused by the procedures involved in the research); and
- Suggests that the research places subjects or others at a greater risk of harm (including physical, psychological, economic, or social harm) than was previously known or recognized.

Relatedness will be determined through the Site PI's chart review and, if necessary, conversations with staff involved in the patient's care to ascertain whether the event was at least possibly related to the study interventions, summarized as "there is a reasonable possibility that the adverse event may have been caused by the trial interventions." Events determined to meet full criteria for unanticipated problems will be reported promptly in accordance with CHOP IRB Policy and NHLBI reporting policy outlined in Section 7 of this Charter. Reports that suggest that subjects or others are placed at a greater risk of harm then initially anticipated will be followed up with an action plan written by the investigators with DSMB consultation.

4. DSMB Membership

DSMB members are listed in the Appendix. The DSMB Chair is responsible for assuring the accurate and timely transmission of the final recommendations. *Ad hoc* members may be added to supplement expertise for single or multiple meetings at the discretion of the Chair.

Proposed DSMB members will undergo conflict of interest review by the Compliance Operations and Conflict of Interest (COI) Department at Children's Hospital of Philadelphia. The investigative team will provide the Department with the names and contact information of proposed DSMB members. Upon receiving this information, the Department will distribute COI disclosure forms to the proposed members for completion. Upon receipt of the forms, the Department will review any potential conflicts and seek clarification from each individual as needed. In accordance with FDA guidance on the structure and operation of clinical trial data monitoring committees, DSMB members should be independent of all entities sponsoring, organizing, conducting, or regulating the trial. They should not have any significant financial interest in the study's conduct or outcome, nor be involved in the study design, nor work at the MPIs' home institutions, nor serve as a Site PI for this trial during their period of DSMB membership. The Department will review COI forms to determine whether any consultancies, relationships with medical device companies, research support, financial interests, or any other relationships exist that could be construed as introducing potential bias to their role as a DSMB member. If any consultancies or financial interests of a proposed member may be viewed as potentially materially impacting their objectivity, they will not be invited to serve on the DSMB. Each DSMB member will be responsible for informing the sponsor and DSMB Chair if any relevant changes in financial interest or other developments affecting potential or perceived conflict of interest develop during the duration of DSMB membership.

The duration of membership for the DSMB is anticipated to be the duration of the trial funding (5 years + a no-cost extension year if needed). In the event that a member withdraws from the Board, the investigators will nominate a replacement in consultation with the DSMB Chair and the NHLBI; the new member will be confirmed at the next scheduled DSMB meeting. Each of the 5 members of the Board will receive $200 per meeting in exchange for their time preparing for and participating in the meeting.

5. Scheduling, Timing, and Organization of Meetings

The purpose of the <u>first meeting</u> is to confirm a Chair, review and discuss this charter, provide an overview of study activities, review and make recommendations about the protocol and informed consent materials, review the reporting templates for data to be presented to the board, and discuss the frequency of interim analyses. Enrollment in a study cannot begin until the DSMB's recommendation for starting is given and IRB approval is granted.

In its <u>subsequent annual meetings,</u> the DSMB will confirm a medical monitor (meeting #2), review outcome data, safety data, racial/ethnic disparity data (with respect to the equity of the effects of the deimplementation strategies on overuse), and subject accrual. Given the seasonal pattern of the trial, with interventions and data collection occurring in winter periods only, we propose convening one DSMB meeting annually with interim analysis planned following each of the 4 winters included in the trial: baseline measurement (Winter 1), active deimplementation (Winter 2), sustainability (Winter 3), and exploratory (Winter 4, when all enrollment will be complete), along with meetings scheduled ad hoc as necessary. An interim analysis will be provided by the Analytic Core after each of the seasons listed above.

The purpose of convening a meeting after Winter 1 is to review data collection numbers, appoint a medical monitor, and make recommendations regarding any changes that should be made to the protocol or recalculation of sample size in the event that data collection numbers are lower than expected.

In addition, the DSMB will, at their discretion, be able to request additional analyses. The DSMB can recommend whether or not to terminate enrollment in the trial because of potential safety concerns or study feasibility issues. At the final meeting following completion of subject enrollment, the DSMB will review final subject accrual and safety data to include in a written report.

The agenda for DSMB meetings and calls may be drafted by the DSMB Scientific Liaison, MPIs, and study biostatistician, and is finalized after consultation with the DSMB Chair. The agenda and meeting materials will be distributed no later than 1 week before each meeting. The NHLBI Program Office may receive this material at the same time as DSMB members.

When the agenda is distributed, DSMB members will be asked to report any new conflicts of interest since the last DSMB meeting. New conflicts will be reviewed by the Chair and study staff to determine if the conflict limits the ability of the DSMB member to participate in the discussion according to conflict of interest policy at Children's Hospital of Philadelphia.

In each DSMB meeting, a study staff member will document meeting minutes summarizing the topics discussed and listing all recommendations. Minutes will be signed by the Chair. After each board meeting, the DSMB Scientific Liaison and MPIs will arrange for a summary of board recommendations to be sent to each participating IRB. In addition, minutes or meeting summaries and the Principal Investigators' follow-up plans will be submitted to NHLBI program staff and the board within 6 weeks following the meeting.

The expertise of the attending members should be appropriate for the agenda of the meeting. It is expected that all DSMB members will attend every meeting. The Chair may have designated replacements for a meeting. For the purposes of voting on recommendations, a quorum is 3 members of the Board.

All standing DSMB members are voting members. The Board may also decide in advance whether ad hoc members can vote.

6. Organization of Meetings

Meetings are organized into open, closed, and executive sessions. They will be held using a virtual platform (e.g. Zoom). The Research Operations Project Manager or an alternate study staff designee will ensure that only the appropriate participants are on the virtual meeting, and invite others to re-join the call only at the conclusion of the executive session.

- Open session – information is presented to the DSMB by the study investigators, with time for discussion. Any proposed changes to the DSMB Charter are discussed. The Open session is attended by the investigative team, study staff, the DSMB and NHLBI program staff. While the trial itself is unblinded, in the open session the aggregate data from the 2 study arms will be presented as "Intervention A" and "Intervention B."

- Closed session – the DSMB, DCC staff, the study staff member documenting minutes, and analytic core staff (including the lead biostatistician) confidentially discuss the material reviewed in the open session in the absence of the investigators. The unblinded trial data are presented by the unblinded statistician in the closed session. NHLBI staff do not attend the closed session unless invited by the Chair to address questions related to the award or about program guidelines.

- Executive session (optional) – This component of the meeting will be limited to DSMB members only, providing the opportunity for independent discussion of all aspects of study progress and drafting of recommendations to the study sponsor, investigators, and funders. NHLBI staff do not participate in executive sessions.

- Recommendations and debrief (optional) – This final component of the meeting will be open to DSMB members, DCC members as appropriate, NHLBI staff, study investigators, and study staff. The DSMB will provide the results of their review and any formal recommendations, which will be discussed as necessary.

7. Adverse Event Surveillance, Reporting, and Management

The risks of this trial are minimal as it involves deimplementation strategies intended to align clinical practice with established evidence, national guidelines, and recommendations for high quality bronchiolitis care. This is primarily an implementation trial, rather than a trial focused on efficacy or effectiveness. The strategies are assigned at the cluster (hospital) level to staff who are caring for patients with bronchiolitis on participating units. In this section we describe potential adverse events in patients that may result from changes in staff physiologic monitoring practices, which may or may not be attributable to the deimplementation strategies. There are no pre-specified stopping rules.

Since the strategies are assigned at the hospital level to staff who are caring for patients with bronchiolitis, we will perform surveillance for adverse events in all bronchiolitis patients hospitalized on units participating in the study. This also includes (a) patients who were not subjects of data collection because the strategies are applied to staff working on participating units and could impact the care of patients with bronchiolitis even if they are not reviewed during data collection rounds, and (b) patients who were indeed subjects of data collection but who may not have had their care impacted in any way by the trial interventions because they are applied to staff members, not directly to patients.

On a monthly basis during each post-randomization winter season (W2, W3, and W4), site investigators will perform surveillance for (a) readmission of bronchiolitis patients within 7 days of discharge from units participating in the trial and (b) code blue and rapid response team activations in bronchiolitis patients hospitalized on units participating in the study that have the potential to meet “unanticipated problems involving risk to subjects” criteria. Using existing local patient safety databases and reports (e.g., local code blue and rapid response team activation logs, readmission reports), site investigators will review the charts of each bronchiolitis patient who was readmitted or was the subject of a code blue or rapid response team call. Readmission reviews will initially determine if the patient was hypoxemic to <85% at the time of re-presentation to the emergency department. Code blue and rapid response team activation reviews will initially determine if the patient was unmonitored and subsequently found to be hypoxemic to <85% at the time of the event when SpO_2 monitors were applied.

Readmissions and code blue/rapid response team activations meeting those initial criteria will be considered study AEs that must be reported, whether or not they are related to the research intervention.

Site investigators will complete a report form administered by the DCC assessing the items in the CHOP IRB “Unanticipated Problems Decision Tree” to analyze relatedness, unexpectedness, and seriousness. It is known that hypoxemia is common in the natural history of bronchiolitis even among children at home and not associated with adverse outcomes in that population (see Principi et al, JAMA Pediatr. 2016;170(6):602-608. doi:10.1001/jamapediatrics.2016.0114). *Therefore hypoxemic events observed during this trial are not necessarily related to the research, unexpected, nor serious, and must be reviewed in the context of this expected natural history.*

A medical monitor will be appointed by the PIs in consultation with the DSMB prior to commencement of the Active Deimplementation study period. The medical monitor may be internal or external to the study team, and may be at an institution where members of the study team are also employed. The medical monitor will assess all Adverse Event and Unanticipated Problem reports sent to the DCC by site investigators, reviewing and electronically signing each report. The monitor will then follow the CHOP IRB “Unanticipated Problems Decision Tree” to appropriately categorize the event and determine the course of action, *keeping in mind the expected natural history of hypoxemia in bronchiolitis and outcomes indicated above based on the Principi study.* Relatedness will be determined through chart review and speaking with staff involved in the patient’s care if the event was at least possibly related to the study interventions, summarized as “there is a reasonable possibility that the adverse event may have been caused by the trial interventions.”

SAE and Unanticipated Problem reporting will adhere to the NHLBI SAE and Unanticipated Problem Event Reporting Timelines illustrated in the table below and available at https://www.nhlbi.nih.gov/grants-and-training/policies-and-guidelines/nhlbi-adverse-event-and-unanticipated-problem-reporting-policy. Investigators must also take into account local IRB guidance if reporting timelines for Unanticipated Problems are shorter than NHLBI Policy. Relying sites' local IRB policies with shorter reporting timelines for SAEs and Unanticipated Problems supersede longer timelines in the NHLBI guidance.

SERIOUS ADVERSE EVENT AND UNANTICIPATED PROBLEM REPORTING TIMELINES

Event Type	**When Event is Reported**<br>*Reconciliation between local IRB Policy and NHLBI Policy based on applying the policy with the shorter reporting timeframe is shown in italics using CHOP as an example. Other relying sites' local IRB reporting requirements may differ. Relying sites' IRB policies with shorter reporting timelines for SAEs and Unanticipated Problems supersede longer timelines in the NHLBI guidance.*	By Whom Event is Reported	To Whom Event is Reported
**Fatal or life-threatening unexpected, suspected serious adverse reactions**	**NHLBI Policy:** Within 7 calendar days of initial receipt of information.<br><br>**CHOP IRB Policy:** SAEs that involve a CHOP subject and involve the death of the subject or are considered life-threatening need to be reported to the IRB within 1 business day of discovery (telephone, fax, email, eIRB) with a full report submitted in eIRB within 48 hours of the initial notification. All unanticipated problems involving subjects external to CHOP must be reported to the CHOP IRB within 7 business days of receipt of the report from the study sponsor, data coordinating center or overall study PI.<br><br>*Policy reconciliation: Refer to CHOP Policy for reporting events involving CHOP subjects to CHOP IRB. Refer to NHLBI policy for all other reporting and reports to CHOP IRB regarding subjects external to CHOP. Relying sites' IRB policies with shorter reporting timelines for SAEs and Unanticipated Problems supersede longer timelines in the NHLBI guidance.*	Investigator<br><br>Sponsor or designee[1]	• Local/internal IRBs<br>• NHLBI and/or Data Coordinating Center (DCC)<br><br>• FDA (if IND study)
**Non-fatal, non-life-threatening unexpected, suspected serious adverse reactions**	**NHLBI Policy:** Within 15 calendar days of initial receipt of information.<br><br>**CHOP IRB Policy:** All other unanticipated problems involving CHOP subjects must be reported within 7 business days of discovery. All unanticipated problems involving subjects external to CHOP must be reported to the CHOP IRB within 7 business days of receipt of the report from the study sponsor, data coordinating center or overall study PI.<br><br>*Policy reconciliation: Refer to CHOP Policy for reporting to CHOP IRB. Refer to NHLBI policy for all other reporting. Relying sites' IRB policies with shorter reporting timelines for SAEs and Unanticipated Problems supersede longer timelines in the NHLBI guidance.*	Investigator<br><br>Sponsor or designee	• Local/internal IRBs/Institutional Officials<br>• NHLBI and/or DCC<br><br>• FDA (IND/Marketed Products)<br>• All participating investigators

**Unanticipated adverse device effects**	**NHLBI Policy:** Within 10 working days of investigator first learning of effect.<br><br>**CHOP IRB Policy:** All other unanticipated problems involving CHOP subjects must be reported within 7 business days of discovery. All unanticipated problems involving subjects external to CHOP must be reported to the CHOP IRB within 7 business days of receipt of the report from the study sponsor, data coordinating center or overall study PI.<br><br>*Policy reconciliation: Refer to CHOP Policy for reporting to CHOP IRB. Refer to NHLBI policy for all other reporting. Relying sites' IRB policies with shorter reporting timelines for SAEs and Unanticipated Problems supersede longer timelines in the NHLBI guidance.*	Investigator<br><br>Sponsor or designee	• Local/internal IRBs<br>• NHLBI and/or DCC<br><br>• FDA (if IDE study)
**Unanticipated Problem that is not an SAE**	**NHLBI Policy:** Within 14 days of the investigator becoming aware of the problem.<br><br>**CHOP IRB Policy:** All other unanticipated problems involving CHOP subjects must be reported within 7 business days of discovery. All unanticipated problems involving subjects external to CHOP must be reported to the CHOP IRB within 7 business days of receipt of the report from the study sponsor, data coordinating center or overall study PI.<br><br>*Policy reconciliation: Refer to CHOP Policy for reporting to CHOP IRB. Refer to NHLBI policy for all other reporting. Relying sites' IRB policies with shorter reporting timelines for SAEs and Unanticipated Problems supersede longer timelines in the NHLBI guidance.*	Investigator	• Local/internal IRBs/Institutional Officials<br>• NHLBI and/or DCC
**All Unanticipated Problems**[2]	**NHLBI Policy:** Within 30 days of the IRB's receipt of the report of the UP from the investigator.	IRB<br><br>Investigator[3]	• OHRP<br><br>• External IRBs

NHLBI Policy Footnotes:
1. Designee is appointed by the sponsor; for example, DCC, CRO.
2. Per OHRP guidance: only when a particular AE or series of AEs is determined to meet the criteria for an UP should a report of the AE(s) be submitted to the IRB at each institution under the HHS regulations at 45 CFR part 46. Typically, such reports to the IRBs are submitted by investigators.
3. Investigators should also take into account local IRB guidance if reporting timelines for UPs are shorter than OHRP guidance

Events that suggest that subjects or others are placed at a greater risk of harm then initially anticipated will be followed up with an action plan written by the investigators with input from the medical monitor and DSMB Chair (and additional members at the discretion of the Chair) that outlines the steps that will be taken to mitigate the newly identified risk(s).

8. Summary Data Reports to the DSMB

For each meeting, the DCC and Analytic Core will prepare summary reports and tables to facilitate the oversight role of the DSMB. The DSMB will discuss at the first or subsequent meetings what data they wish to review and how it should be presented. Reporting templates will be included in the minutes of the first DSMB Meeting unless otherwise specified by the Board.

9. Board Recommendations

Board Recommendations signed by the DSMB Chair, will be sent to the investigators within 14 calendar days after the meeting. Recommendations should include a statement as to whether the study is approved to continue as planned, should continue with specified changes, or should be stopped. Requests for additional data from the investigators or DCC/analytic core should include an expected due date. In addition to recommendations memos issued to investigators (for review and IRB distribution), recommendations related to data or data analysis issues may be issued separately for DCCs or statisticians.

Recommendations are distributed by the principal investigator to each clinical center, the DCC, and the NHLBI Program Office. It is the responsibility of each clinical center to forward this information to their local IRB.

10. Statistical Monitoring Guidelines

At the first meeting, review of the protocol will include review of the statistical analysis plan (a section of the IRB protocol). The DSMB will discuss the adequacy of that plan. The DSMB will discuss the statistical monitoring procedures they propose to follow to guide their recommendations about termination or continuation of the trial. These procedures will be documented in the DSMB meeting minutes.

11. Appendix: DSMB Members

Name and Email Address	Specialty and Affiliations
Sanjay Mahant, MD, MSc, FRCPC **(Chair)** sanjay.mahant@sickkids.ca	*Pediatric Hospital Medicine* Staff Paediatrician, The Hospital for Sick Children Professor, Paediatrics, University of Toronto Chair, Pediatric Inpatient Research Network (PIRN), Canada
Ted A. Skolarus, MD, MPH, FACS tskolar@med.umich.edu	*Implementation Science* Surgery Service Chief, VA Ann Arbor Healthcare System Associate Professor of Urology, University of Michigan Research Scientist, VA HSR&D Center for Clinical Management Research
Mary Sammel, ScD mary.sammel@cuanschutz.edu	*Biostatistics* Professor of Biostatistics, Colorado School of Public Health, University of Colorado Associate Director, Center for Innovative Design & Analysis, Department of Biostatistics & Informatics, University of Colorado
David Stockwell, MD, MBA stockwell@jhu.edu	*Pediatric Critical Care Medicine* Chief Medical Officer, Johns Hopkins Children's Center Associate Professor of Anesthesiology and Critical Care Medicine, Associate Professor of Pediatrics, Johns Hopkins University
Ian Wolfe, PhD, MA, RN, CCRN, HEC-C wolfe370@umn.edu	*Clinical Ethics, Nursing* Staff Clinical Ethicist, Children's Minnesota Adjunct Faculty, Center for Bioethics, University of Minnesota Editor-in-Chief, Journal of Pediatric Ethics
**NHLBI Invitees**	
Aruna Natarajan, MD, PhD, FAAP aruna.natarajan@nih.gov	Program Director, NHLBI Pediatric and Neonatal Lung Disease and Critical Care
Karen Bienstock, PA-C karen.bienstock@nih.gov	Clinical Trials Specialist, NHLBI Division of Lung Diseases
