# Supplementary material for: Sustainable deimplementation of continuous pulse oximetry monitoring in children hospitalized with bronchiolitis: study protocol for the Eliminating Monitor Overuse (EMO) type III effectiveness-deimplementation cluster-randomized trial
Source: Implement Sci. 2022 Oct 21;17:72. doi: 10.1186/s13012-022-01246-z (PMC9587657; doi:10.1186/s13012-022-01246-z)
Supplement: Supplementary file 2 — Additional file 2. Dissemination plan for results of the EMO Trial. Description: Description of the plan for disseminating results and products of the EMO trial. This document was also submitted with the EMO Trial grant proposal. [file 13012_2022_1246_MOESM2_ESM.pdf]

EMO TRIAL DISSEMINATION PLAN

The proposed trial will be registered with clinicaltrials.gov by the study MPIs prior to subject enrollment in a manner similar to our pilot study (ClinicalTrials.gov identifier: NCT04178941). Results information will be submitted to clinicaltrials.gov within 12 months after the primary completion date, in accordance with the NIH Policy on the Dissemination of NIH-Funded Clinical Trial Information.

The informed consent documents will include a specific statement that aggregate data from the clinical trial will be posted to clinicaltrials.gov in compliance with government policies and the internal policies of the Children's Hospital of Philadelphia.

Informed consent documents will include the following statement in compliance with government policies and the internal policies of the Children's Hospital of Philadelphia. This is standard institutional language posted on the Children's Hospital of Philadelphia IRB website.

"A description of this clinical trial will be available on http://www.ClinicalTrials.gov, as required by U.S. Law. This Web site will not include information that can identify you. At most, the Web site will include a summary of the results. You can search this Web site at any time."

The Children's Hospital of Philadelphia Office of Research Compliance has an internal policy in place to ensure that clinical trials registration and results reporting occur in compliance with policy requirements.

Results will also be submitted for publication in a peer-reviewed journal not later than one year after the trial's primary completion date. We have budgeted for publication fees in order to make the results available to the public in an open access form as quickly as possible.

In addition to clinicaltrials.gov and submission for peer-reviewed publication, we will present the results in scientific meetings such as Pediatric Academic Societies, the Pediatric Hospital Medicine Annual Meeting, the American Thoracic Society, and the Annual Conference on the Science of Dissemination and Implementation in Health (D&I) co-hosted by the National Institutes of Health and AcademyHealth.

Electronic health record implementation guides and clinical decision support content will be disseminated freely to the public via the AHRQ CDS Connect Repository and EHR-specific community libraries, if the EHR intervention leads to effective deimplementation.

As an additional dissemination activity, we will convene an in-person meeting in Year 5 of the award among the investigators, PRIS Network leadership, nurses and physicians from PRIS hospitals, the national Pediatric CDS Collaborative, and the Advisory Committee (which includes American Academy of Pediatrics leaders) to plan for widespread dissemination of the results to the pediatric community at large.
